# Time Management Strategies of Rock Climbers in World Cup Bouldering Finals

**DOI:** 10.5114/jhk/159652

**Published:** 2023-01-20

**Authors:** Ben J. Mckellar, Alexandra M. Coates, Jeremy N. Cohen, Jamie F. Burr

**Affiliations:** 1 Department of Human Health and Nutritional Science, University of Guelph, Guelph, ON, Canada.

**Keywords:** slab, coordination, zones, attempts, success rate, tops

## Abstract

Competitive rock climbing recently made its Olympic debut, but minimal published research exists regarding training and competition strategies. Time management strategies define the structured approach climbers take in bouldering competitions to successfully obtain a “top” or a “zone” hold. During finals rounds of the International Federation of Sport Climbing bouldering competitions, climbers are allotted 240 s to complete a boulder. Variables influencing a climber’s time management strategies include their work-to-rest intervals, and the frequency of their attempts or rests. Video analysis of International Federation of Sport Climbing competitions was used to collect time management strategy data of professional climbers. Fifty-six boulders (28 female and 28 male boulders) over the 2019 International Federation of Sport Climbing season were analyzed. Time management strategies variables were compared between slab/slab-like and non-slab bouldering styles using generalized estimating equations with significance set to p < 0.05. Additionally, we determined trends in success rates for various styles of boulders. There were no differences in the number of attempts taken per boulder between slab/slab-like and non-slab boulders (3.7 ± 2.3 and 3.8 ± 2.4, p = 0.97), but climbers spent more time actively climbing on slab/slab-like (92 ± 36 s) compared to non-slab boulders (65 ± 26 s, p < 0.001). Trends in the success rate suggest climbers who take more than 6 attempts on any boulder style are unsuccessful. The results of this study provide practical information that can be used by coaches and athletes to guide training and competition strategy.

## Introduction

The popularity of rock climbing is rapidly increasing with mainstream attention, and with its inclusion in the recent Tokyo Olympic Games, it seems likely interest in the sport will continue to grow. Competition climbing is comprised of three disciplines, including speed, bouldering, and lead climbing. Bouldering requires no use of a rope or harness and occurs on a shorter wall (maximum 4 m) with a padded mat to cushion climbers if they fall. The sequence of holds on a boulder route is always unique (Hatch and Leonardon, 2020). Analysis of the characteristics of speed and lead climbing competitions have been studied previously (Arbulu et al., 2015; Guo et al., 2019); however, the current analysis specifically offers insight into the bouldering discipline.

The structure of a bouldering finals round begins with a two-minute preview period for each boulder. There are four boulders within a finals round, with 240 s of allotted time to complete each boulder (Hatch and Leonardon, 2020). Unlike qualification and semi-final rounds, there is no standard allotted time to rest between boulders in the finals, as it is dependent on how quickly the other competitors complete the boulder. As such, time management strategies (TMS) during the 240 s become an important consideration for performance in the finals rounds.

TMS are the structured approach climbers take in competition to successfully complete one or more segments of a climb within their allotted 240 s. These TMS incorporate interactions between work-to-rest intervals, and the quantity of attempts and rests a climber takes within their allotted time. Points are awarded when a climber uses a marked hold within the boulder, and when they finish the boulder, known as “zones” and “tops”, respectively. A top in bouldering is given when a climber has secured the final demarcated hold, successfully completing the boulder. A zone is a hold approximately midway up the boulder used to differentiate scores if climbers are tied based on the number of tops and attempts to obtain a top.

Previous work has studied climbers during preliminary rounds and simulated competitions in bouldering (Augste et al., 2021; Künzell et al., 2020; Medernach et al., 2016; White and Olsen, 2010). Those studies have assessed characteristics related to the number of attempts and climbing time within a climbing period, in addition to individual mean attempt and rest times, and attempt times when a boulder was completed. Limited research has investigated the bouldering style and how these characteristics influence TMS (Augste et al., 2021; Medernach et al., 2016). Determining differences in TMS of bouldering styles is important for competition and training strategies, as the physiological requirements and preparation may be different. Considering the general climbing literature, an increased wall gradient can differentiate the climbing ability level on a time-to-fatigue task (Baláš et al., 2021), due to greater force application through the hands and fingers relative to the feet (Noé et al., 2001), increased core muscle activation (Park et al., 2015), increased blood lactate accumulation, and decreased total distance climbed (Fryer et al., 2011; Watts and Drobish, 1998). The primary limiting factor to rock climbing performance is fatigue of the finger flexors (Deyhle et al., 2015); therefore, increased reliance on the upper body and hand gripping musculature with increased wall steepness provides a strong rationale for altered TMS between climbing styles/wall angles.

The aims of this investigation were to 1) characterize a typical bouldering finals round in an elite competition setting, (2) elucidate the value of TMS in successfully obtaining a top or a zone hold to develop recommendations for training and competition strategies, and (3) determine if there are differences in TMS between particular climbing styles.

## Methods

### Participants

Twenty males and eighteen females who made the finals rounds over seven International Federation of Sport Climbing (IFSC) bouldering competitions in 2019 were included in this study. A total of 42 possible finals competitor positions were available over the seven competitions for both men and women, indicating some competitors made multiple finals appearances over the IFSC season.

### Measures

Data collection was accomplished through video analysis using an online repository where all videos had been posted publicly. As such no ethics approval was required. Performance variables taken directly from the video recordings were the time when an attempt started or finished, which was also the time at which a rest interval finished and started. The total number of attempts a climber took within a bouldering period and the attempt number at which the climber obtained a zone hold or a top were recorded. The remainders of performance variables were determined from the values taken from the video recording and are shown in Table 1.

### Design and Procedures

In the 2019 IFSC Bouldering World Cup season, there were seven bouldering competitions (Meiringen, Switzerland; Moscow, Russia; Chongqing, China; Wujiang, China; Munich, Germany; Vail, USA; and Hachioji, Japan), with four finals boulders for both male and female competitors in each of the seven competitions. Therefore, 56 unique boulders (28 male and 28 female) and 336 climbing periods (7 world cups x 6 athletes x 4 boulders x 2 sexes) were included in analysis and data collection.

### Slab/Slab-Like, Non-Slab and Combined Styles Definitions and Criteria

**Figure 1:**
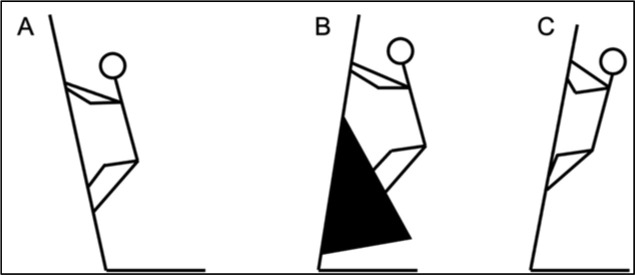
Illustration of bouldering styles included in analysis. A) Slab, wall angle >90 degrees from horizontal. B) Slab-like, wall angle <90 degrees from horizontal, but large volumes (solid black triangle) alter the climber’s position to mimic slab. C) Non-slab, wall angle <90 degrees from horizontal.

The generally agreed upon definition of “slab” is a wall angle greater than 90 degrees from horizontal (Figure 1A). We also defined “slab-like” bouldering styles (Figure 1B), which include boulders with wall angles slightly less than 90 degrees from the ground, but use large volumes or wall features (black triangle in Figure 1B) to change the climber’s position relative to the wall. For this analysis, boulders were categorized as the slab-like style when climbers spent a large majority of the boulder pushing on holds as opposed to pulling on them, or when climbers were capable of being hands-free on a boulder in the absence of any obvious steep wall angles. Slab and slab-like boulders were included in the same group for analysis and referred to as slab/slab-like (SSL). Non-slab (NS) boulders (Figure 1C) are boulders with wall angles less than 90 degrees from horizontal that did not fall into the slab-like category. Some boulders were excluded from SSL and NS styles as they had characteristics of both styles, but these boulders were included in the “combined styles” category, which included all boulders in all seven competitions. A complete outline of the competitions and boulders that were included in the NS and SSL groups is presented in Supplementary Table 1.

**Table 1:** Variables included in analysis from live streamed IFSC bouldering world cup finals events and their calculations.

Variable	Calculation
Attempt time	Video time at the end of an attempt – video time at the start of an attempt
Rest time	Video time at the end of rest – video time at the start of rest
Cumulative attempt time	Sum of attempt times in each climbing period
Cumulative rest time	Sum of rest time in each climbing period
Total boulder attempt number	No calculations. Number of attempts taken by a climber in a climbing period
Total boulder rest number	No calculations. Number of rests taken by a climber in a climbing period
Top attempt time	Video time at the end of an attempt when the boulder was topped – video time at the start of an attempt when the boulder was topped
Cumulative attempt time with no top	Sum of attempt times in a climbing period when the climber did not top the boulder
Total boulder attempt number with no top	No calculations. Number of attempts taken by a climber in a climbing period when no top was obtained
Cumulative attempt time before a top	Sum of attempt times up to and including the top attempt
Attempt number to obtain a top	No calculations. The attempt number in a climbing period at which a top was obtained
Attempt number to obtain a zone	No calculations. The attempt number in a climbing period at which a zone was obtained
Cumulative competition attempt time	Sum of attempt times for an individual climber over all boulders in a single competition

### Coordination Moves

A secondary analysis was performed characterizing a third style of climbing termed “coordination moves”. Currently, there is no specific definition of a coordination move in the rock-climbing literature; however, it is understood by coaches, athletes, and route setters that these types of moves involve a complex sequence of limb movements performed in fast succession by the climber. For this study, a coordination move was defined as requiring at least three limb movements (either foot or hand) each contacting a hold(s) in fast succession occurring in less than 2 s. Additionally, the majority of climbers must have attempted the boulder using the coordination move for the boulder to be included in analysis. In some instances, the majority of climbers figured out a sequence of moves to avoid the intended coordination move, thus, excluding the boulder from being categorized as such. Coordination moves occur within a boulder, thus, the completion of a coordination move was independent of completion of the boulder. This style of climbing was important to include within the analysis as according to our definition, 18% of boulders included in analysis contained a coordination boulder. This indicates that at the professional level, climbers are required to be proficient in this style of climbing to be successful. A list of boulders with coordination moves included in analysis can be found in Supplementary Table 2.

**Table 2:** Time management and climbing performance measures during slab/slab-like, non-slab and combined style boulders during IFSC bouldering finals events in the 2019 competition season.

Variable	Combined		Non–Slab		Slab/Slab–like		GEE sig
	*Mean ( ± SD)*	*Range*	*Mean (± SD)*	*Range*	*Mean ( ± SD)*	*Range*	
Attempt time	19 ± 18	1–125	17 ± 16	1–120	25 ± 20*	1–125	**<0.001**
Rest time	28 ± 18	1–140	30 ± 20**	2–140	22 ± 12	3–61	**<0.001**
Cumulative attempt time	72 ± 32	14–177	65 ± 26	14–143	92 ± 36*	33–177	**<0.001**
Cumulative rest time	94 ± 45	6–185	105 ± 43**	11–185	72 ± 36	6–133	**<0.001**
Total boulder attempt #	3.7 ± 2.4	1–13	3.8 ± 2.4	1–13	3.7 ± 2.3	1–10	0.966
Attempt time of a top	40 ± 18	14–125	39 ± 17	14–120	45 ± 20	20–125	0.114
Cumulative attempt time with no top	88 ± 31	22–177	78 ± 22	22–141	119 ± 28*	71–177	**<0.001**
Total boulder attempt # with no top	5.4 ± 2.2	2–13	5.2 ± 2.1	2–13	5.4 ± 2.2	2–10	0.636
Cumulative attempt time before a top	58 ± 26	14–143	52 ± 24	14–143	70 ± 27*	33–135	**<0.001**
Attempt # to obtain a top	2.3 ± 1.4	1–9	2.1 ± 1.4	1–9	2.4 ± 1.4	1–6	0.167
Attempt # to obtain a zone	2.6 ± 1.9	1–8	2.6 ± 2	1–8	3.0 ± 1.6	1–7	0.343
Cumulative competition attempt time	289 ± 70	140–466					

All variables related to time are described in seconds. * Significantly greater than non-slab, ** significantly greater than slab/slab-like (p < 0.05).

### Definition of an Attempt and Obtaining a Zone Hold

We defined the start of an attempt as the point at which no part of the climber’s body was touching the ground; whereas an attempt ended when the climber first contacted the mats after falling from the boulder. We referred to the IFSC rules for the definition of “control” of a zone hold (Hatch and Leonardon, 2020). Briefly, a climber must “use” the zone hold, not simply touch it. Data for zones were collected only if a top was not obtained. According to the IFSC, a hierarchy of scoring is used whereby climbers are initially scored based on the number of tops achieved in a round, followed by the number of attempts to obtain a top, the number of zones, and the number of attempts to obtain a zone (Hatch and Leonardon, 2020). Therefore, zones are typically only important if a climber has not achieved a top.

### Statistical Analyses

Descriptive statistics were produced for TMS variables and are presented in Table 1 for SSL, NS, and combined styles. The success rate (defined below) was calculated for SSL, NS, combined styles, zones, and coordination moves for each attempt number. Success indicates a climber had successfully topped a boulder, controlled a zone hold, or completed a coordination move. For each attempt, the success rate was calculated as the number of successful climbers / the number of climbers who took that attempt. These calculations were performed for all attempts (1 through 13, with 13 being the maximum taken by any climber). Each attempt was characterized as either successful or unsuccessful, therefore the number of eligible climbers decreased with increasing attempts, as successful climbers were not required to continue. Therefore, the success rate is the relative chance of success during each attempt, whereas the number of successful climbers is the absolute value changing with each attempt.

Due to the nature of our data set, and the fact that multiple climbers competed in various numbers of competitions (shown in supplementary Table 3), traditional parametric statistics were inappropriate and general estimating equations (GEEs) were computed instead. Two-tailed linear GEE models were performed with climbers as the repeated measure, climbing style (SSL or NS) as the predictor variable, and climbing outcomes (Table 1) as dependent variables. All statistics were performed using SPSS version 28 (IBM, USA) with alpha set *a priori* at *p* < 0.05.

## Results

### TMS Strategies of Climbers

Performance variables including the total boulder attempt number, total boulder attempt number with no top, number of attempts to obtain a zone, and the number of attempts to obtain a top did not differ between SSL and NS styles. Conversely, most variables related to time, including attempt time, rest time, cumulative boulder attempt time, cumulative boulder rest time, cumulative boulder attempt time with no top, and cumulative boulder attempt time before a top, were significantly different between SSL and NS styles. Attempt time of a top was the only exception as it was not significantly different between styles. During SSL boulders, climbers took longer attempts, but rested for less time between those attempts compared to NS as shown in Table 2.

### Trends in Success with the Increasing Attempt Number

**Figure 2:**
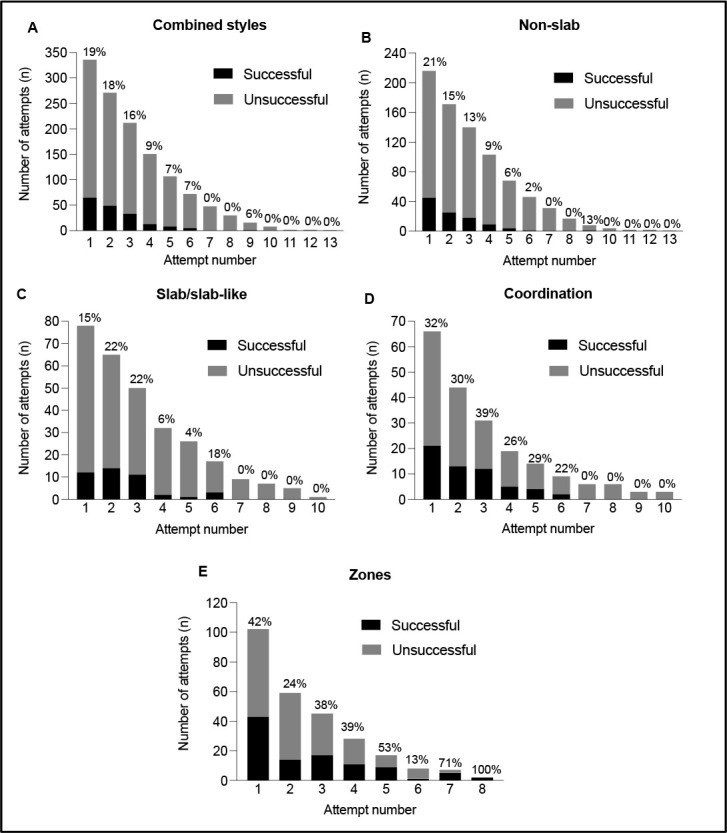
Trends in the success rate and the number of successful climbers as the attempt number Increases. The y-axis values represent the absolute change in the number of climbers taking an attempt. The percentage above the bars represents the relative success rate for each attempt number where black bars are the number of successful climbers that would not be taking any further attempts on the boulder after completion.

**Figure 3:**
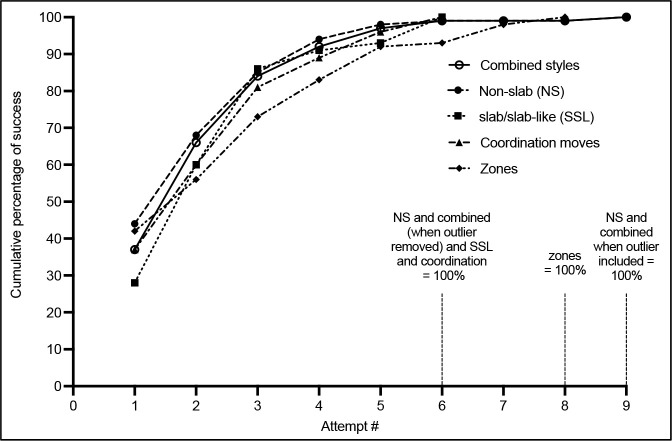
Trends in the cumulative percentage of successes as the attempt number increases in various bouldering styles. The 100% value indicates the attempt number at which all climbers within a bouldering style had their successful events occur.

There were a total of 290 attempts of SSL-style boulders, and 809 attempts of NS-style boulders. Climbers were successful 14.8% and 12.9% of the time in SSL and NS styles, respectively. Competitors took 201 attempts on coordination moves with a success rate of 28.4%. There were 268 attempts to obtain zone holds in the absence of a top, and 38.1% of these attempts resulted in a climber obtaining a zone. Data on the success rate and cumulative percentage of tops for increasing attempt numbers are shown in Figures 2 and 3, respectively. In the combined and NS styles of Figure 2 and 3, only a single climber was successful on their ninth attempt. This individual data point was a definitive outlier and will not be considered in the discussion of success trends, however, it does demonstrate the possibility of a rare occurrence and is reported as such.

The cumulative percentage of success describes the progression of total tops over successive attempts, which was different between styles. As can be seen in Figure 3, after attempt one the percentage of total tops ranged from 28% in SSL to 44% in NS. By attempt three, NS and SSL styles were within 1% of each other. Apart from attempt one, the rate of an increase in coordination moves and obtaining zone holds progressed at a lower rate compared to the other styles.

## Discussion

The novel findings of this research on IFSC bouldering competitions are that time management strategies during SSL and NS style boulders differed, as SSL boulders required longer attempts and climbers spent less time resting compared to NS. Despite this, in both styles of bouldering the quantity of attempts required was similar. We also found that irrespective of the boulder style, the success rate after the 6^th^ attempt was zero (excluding the outlier), and the relationship between the success rate with increasing attempts was different between SSL and NS-style boulders. There was a steep decline in success following the first attempt in NS-style boulders, but a more stable success rate during the first three attempts of SSL-style boulders.

### TMS Strategies of Climbers

Previous literature examining TMS in bouldering competitions demonstrates a range of 2.8–5.1 attempts per boulder period (Callender et al., 2020; Künzell et al., 2020; Medernach et al., 2016; White and Olsen, 2010), with 64–92 s of cumulative active climbing time per boulder (La Torre et al., 2009; Medernach et al., 2016; White and Olsen, 2010). Attempt time ranged between 15 and 30 s (Medernach et al., 2016; White and Olsen, 2010), with 27–115 s of rest between attempts (Medernach et al., 2016; White and Olsen, 2010), and attempts’ duration of 34–41 s when the boulder was topped (Medernach et al., 2016; White and Olsen, 2010). All values in the present study in the combined styles group fell within the existing mean ranges reported above, despite some differences in competition rules related to allotted climbing time and rest between boulders.

Climbers generally took a similar number of attempts within the allotted 240 s period between NS and SSL styles, but the work-to-rest interval within that period differed. During the SSL boulders, climbers spent longer climbing per attempt and took shorter rest periods between attempts compared to NS. It is possible that this relates to the differing physiological demands posed by wall inclination. Steeper wall angles require greater reliance on finger, wrist and forearm flexor musculature (Baláš et al., 2014; Fryer et al., 2011; Noé et al., 2001; Park et al., 2015; Watts and Drobish, 1998). As such, the NS style likely increases the rate and magnitude of muscular fatigue compared to SSL, forcing climbers to have shorter attempts and take longer rest periods following those attempts. Considering that forearm muscle fatigue is a limiting factor for climbing performance (Deyhle et al., 2015), it is not surprising that climbers would rest for longer periods if they felt significant fatigue. Optimizing this recovery period between exhaustive bouts on a boulder could be a vital focus for training.

### Trends in Success with an Increasing Attempt Number

In the SSL style, the success rate (relative chance of success during each attempt) and the number of successful climbers (absolute change in successful climbers with each increasing attempt) increased from attempt one to attempt two and remained elevated in attempt three. This contrasts with NS, which showed a steady decrease in the success rate and the absolute number of successful climbers following attempt one. The differing physiological requirements described above offer a strong rationale for these differences, but the technical requirements between these styles are also an important consideration. Although no research is currently available, SSL style boulders are thought to require precise and controlled movements, highly dependent on the body position and location of the center of gravity relative to the wall. The increased success rate and the absolute number of successful climbers during later attempts in SSL boulders could be due to a practice or learning effect during the specific boulder. The first and second attempts on SSL boulders could be used to make small adjustments in body positions from the previous attempt(s), without a significant accumulation of fatigue, to allow climbers to better learn the sequence of movements to progress through the boulder. This is also true of coordination moves, which demonstrated the greatest success rate at attempt three. While the practice effect is still present in NS boulders, the development of fatigue could outweigh the benefits of learning from a previous attempt(s). These findings are further emphasized with the cumulative percentage of success, wherein after attempt one, the cumulative percentage was lowest in SSL boulders and highest in NS boulders, but the magnitude of difference quickly equilibrated in the following attempts. Despite more climbers being successful at obtaining a zone on their first attempt than subsequent attempts, the success rate was highly variable in the following attempts. It is difficult to make any clear conclusions for TMS strategies relating to zones. It does appear that additional attempts after attempt six may be warranted to obtain a zone considering that the cumulative percentage of success takes more attempts relative to other styles, and the success rate is still elevated.

TMS of the NS boulder style are applicable for competition-specific muscular strength and anaerobic capacity training, as physiological demands appear greater in this style compared to SSL. Based on the present findings, coaches and athletes should use a variety of work-to-rest ratios, and structure these into a typical 4- to 5-min period adjusting intensity and volume using the means ± SDs in Table 2. In training, simulating situations wherein climbers are struggling to obtain a top within their allotted time (i.e., using the variables in Table 2 where climbers were unable to obtain a top) may be advised to ensure climbers are prepared for the potential fatigue development within a bouldering round. Climbers spent 289 ± 70 s actively climbing in boulder finals rounds thus, this active climbing time can be used to determine the volume of work performed in training sessions. The results of this study can be used to guide the decision making of an athlete during a bouldering period within a competition, although it is still essential for the athlete to understand and consider their own levels of fatigue. For example, our data indicate that after the fourth attempt on a NS boulder, the success rate on the fifth attempt decreases to 6%. The athlete can use this knowledge, in addition to their current level of fatigue, to decide whether they should take another attempt on the current boulder or preserve energy for future boulders in the round. For obtaining a top, climbers should not take greater than six attempts regardless of the bouldering style, but this does not, however, mean a climber should take six attempts on every boulder. As this study cannot determine what the ideal attempt number should be, six attempts should be considered the absolute cut-off for obtaining a top. It is pertinent that climbers prioritize their first attempt, specifically with NS style boulders, as it will be their greatest chance for success. Finally, if a climber is unable to obtain a zone by their sixth attempt during a competition, additional zone-attempts may be warranted

Although this study is the most comprehensive for identifying TMS in professional climbers to date, it is not without limitations. Only climbers who made finals were included, due to limitations of live streamed video not allowing for adequate video analysis of qualification and semi-final rounds. It could, however, be argued that competitors who made finals rounds would tend to possess better TMS in addition to skill. It should be noted that skill likely causes some skew in the success rate results. Better climbers are, by definition, required to be successful on earlier attempts and will take part in a greater number of competitions, therefore increasing the absolute number of successful climbers on earlier attempts and increasing the success rate. This was accounted for within our GEE modeling of TMS variables, but not possible for our success rate figures. We acknowledge this limitation and intentionally phrase our practical application recommendations to indicate we cannot determine what the optimal attempt number is, but instead, we used the absolute cut-offs for success as our recommendation.

Future research should additionally identify TMS in qualification and semi-finals rounds and determine if these TMS in previous rounds can predict placement in future rounds. For example, assessing the relationship between total active climbing time in the qualification round with placement in the semi-finals round, and similarly for the semi-finals to finals. Similar investigations should also occur in different climbing populations (e.g., youth categories, sub-elite) and competition formats.

Herein we present the most comprehensive study to date assessing bouldering competition TMS. We provide evidence that the TMS approaches climbers take during NS and SSL style boulders are different, indicating training for climbing competitions should not be uniform. Finally, we present data on the success rate of increasing attempts and cumulative percentage of successes over increasing attempts that can help guide training and coaching practices to improve athletes’ competition strategy and performance.
